# Comparison of the protective efficacy between single and combination of recombinant adenoviruses expressing complete and truncated glycoprotein, and nucleoprotein of the pathogenic street rabies virus in mice

**DOI:** 10.1186/s12985-017-0789-2

**Published:** 2017-06-24

**Authors:** Ha-Hyun Kim, Dong-Kun Yang, Jin-Ju Nah, Jae-Young Song, In-Soo Cho

**Affiliations:** Viral Disease Division, Animal and Plant Quarantine Agency, Gimcheon, 39660 MAFRA Republic of Korea

**Keywords:** Human adenovirus, Pathogenic street rabies virus, Glycoprotein, Nucleoprotein

## Abstract

**Background:**

Rabies is an important viral zoonosis that causes acute encephalitis and death in mammals. To date, several recombinant vaccines have been developed based on G protein, which is considered to be the main antigen, and these vaccines are used for rabies control in many countries. Most recombinant viruses expressing RABV G protein retain the G gene from attenuated RABV. Not enough is currently known about the protective effect against RABV of a combination of recombinant adenoviruses expressing the G and N proteins of pathogenic street RABV.

**Methods:**

We constructed a recombinant adenovirus (Ad-0910Gsped) expressing the signal peptide and ectodomain (sped) of G protein of the Korean street strain, and evaluated the immunological protection conferred by a single and combination of three kinds of recombinant adenoviruses (Ad-0910Gsped and Ad-0910G with or without Ad-0910 N) in mice.

**Results:**

A combination of Ad-0910G and Ad-0910 N conferred improved immunity against intracranial challenge compared to single administration of Ad-0910G. The Ad-0910G virus, expressing the complete G protein, was more immunogenic than Ad-0910Gsped, which expressed a truncated G protein with the transmembrane and cytoplasmic domains removed. Additionally, oral vaccination using a combination of viruses led to complete protection.

**Conclusions:**

Our results suggest that this combination of viruses is a viable new intramuscular and oral vaccine candidate.

## Background

Rabies virus (RABV) is an important causative agent of viral zoonosis resulting in acute encephalitis and death in mammals. RABV belongs to the genus *Lyssavirus* in the *Rhabdoviridae* family, and contains a single-stranded, non-segmented, negative-sense RNA of about 12 kb [1]. RABV consists of ribonucleoprotein (RNP), which is made up of nucleoprotein (N), phosphoprotein (P), large polymerase protein (L), and viral genomic RNA, as well as a virion lipid envelope containing matrix protein (M) and glycoprotein (G) surrounding the RNP [1, 2]. Among these proteins, the G and N proteins are known to be important for immunogenicity against RABV. The G protein is the major antigen in the formation of neutralizing antibodies (Nab) against RABV in animals [3–5]. The N protein has more stable antigenic, immunologic, and genetic properties than the G protein, and stimulates production of protective antibodies against rabies. Therefore, N protein is considered an alternative candidate immunogen against RABV [1, 6–9].

Currently, most recombinant vaccines against rabies are based on the G protein because this protein has been considered the main antigen for the formation of Nab. The vaccinia-rabies glycoprotein (V-RG) recombinant virus vaccine contains recombinant vaccinia virus (Copenhagen strain) expressing the G protein of the Evelyn-Rokitnicki-Abelseth (ERA) strain and is the first licensed recombinant poxvirus vaccine [10, 11]. The V-RG vaccine has been shown to induce protective immunity in dogs, mice, and rabbits by intradermal immunization, raccoons by oral vaccination, and skunks by the bait-feed, intestinal, intramuscular, and scarification routes [4, 12–14]. The V-RG vaccine has been used as an oral vaccine in red foxes in several western European countries, raccoons, gray foxes, and coyotes in North America, raccoons in Canada, and raccoon dogs in Korea [11, 12, 15, 16]. Several studies have described human adenovirus recombinants expressing the rabies G protein [17–19]. The first construct, AdRG1, was developed by inserting the rabies G gene from the ERA strain into the E3 region [17, 19]. A second construct, AdRG1.3, referred to as ONRAB, induced effective immunity via the oral route in skunks and raccoons [18–20]. Recombinant canine adenoviruses expressing the RABV G protein from SAD B19 RV strain and vaccine strain SRV_9_ have been found to be immunogenic in mice, dogs and sheep [21–24]. Most recombinant viruses expressing the RABV G protein retain the G gene from attenuated and fixed strain such as the ERA.

There has been insufficient information about protection against RABV using recombinant virus expressing the G protein of pathogenic street RABV and in combination with recombinant virus expressing N protein from pathogenic virus. Therefore, we reported safety and immunogenicity of combined injection of recombinant human adenoviruses (Ad-0910G and Ad-0910 N) expressing the G and N proteins of the Korean street strain (KRVB0910) in Korean raccoon dogs via intramuscular and oral administration in the previous study [25]. In this study, we constructed a recombinant adenovirus (Ad-0910Gsped) expressing the signal peptide and ectodomain (sped) of G protein and evaluated the protective immunity induced by a single use and combination of recombinants adenoviruses (Ad-0910Gsped and Ad-0910G with or without Ad-0910 N) against intramuscular and intracranial challenge in mice.

## Methods

### Cells and viruses

293A cells (human embryonic kidney cells transformed with the E1 region of human adenovirus type 5) were maintained in Dulbecco’s modified Eagle’s medium (DMEM) supplemented with 10% FBS, 2 mM L-glutamine, 0.1 mM MEM non-essential amino acids (NEAA), 100 U/mL penicillin, and 100 μg/mL streptomycin. NG108–15 cells were grown in DMEM supplemented with 10% FBS, 100 U/mL penicillin, and 100 μg/mL streptomycin in a 5% CO_2_ humidified incubator. Korean rabies isolate KRVB0910 was isolated from a brain sample from RABV-infected cattle in Goseong, Gangwon province in 2009. The RABV ERA strain was propagated in NG108–15 cells as a positive control for western blot analysis. The CVS-N2c strain of rabies virus was propagated in the brains of newborn mice and the median lethal dose (LD_50_) was determined by titration in adult mice.

### Amplification of signal peptide and ectodomain (sped) of G gene

Total RNA was extracted from the brain sample using the Qiagen RNeasy mini kit (Qiagen) according to the manufacturer’s instructions. The signal peptide and ectodomain (sped) of G gene of Korean rabies isolate KRVB0910 (GenBank accession no. KJ476819) were amplified by RT-PCR using specially designed primer pair. The primer sequences are as follows: sped of G, forward 5′-CACC ATG GTT CCT CAG GCT CTC CT-3′, reverse 5′-TCA CTC CCC CCA GTT AGG GAG AC-3′. RT-PCR was conducted in a reaction mixture containing 5 μL of denatured RNA, 1.5 μL of each primer (10 μM), 5 μL of 10× buffer, 1.5 μL of dNTP mix (10 mM), 1 μL of MgSO_4_ (50 mM), 0.4 μL of Platinum *Pfx* DNA polymerase (Invitrogen), 1 μL of AMV reverse transcriptase (Promega), 1 μL of ribonuclease inhibitor (Promega), and 32.1 μL of distilled water. The cycling profile consisted of cDNA synthesis at 42 °C for 30 min, followed by 35 cycles of 94 °C for 30 s, 55 °C for 30 s, and 68 °C for 1 min, with a final extension at 68 °C for 5 min. The PCR products were visualized using electrophoresis on 1.8% agarose gels containing ethidium bromide.

### Construction of recombinant adenovirus

Recombinant human adenoviruses (Ad-0910G and Ad-0910 N) expressing the complete G and N proteins of KRVB0910 were constructed in the previous study [25]. Recombinant adenovirus expressing the sped of G protein was constructed using the ViraPower adenoviral expression system (Invitrogen) according to the manufacturer’s instructions. Briefly, the amplified RT-PCR product was cloned into the entry vector of the adenoviral expression system to construct the recombinant entry plasmids pENTR-Gsped. Adenoviral expression clone was generated by performing an LR recombination reaction between the entry plasmid and replication-deficient, E1-deleted human adenovirus (Ad) type 5 pAd-DEST vector. The generated adenoviral expression clones (pAd-0910Gsped) was digested with *Pac*I, and then transfected into 293A cells. After CPE appearance, cell culture was harvested and propagated in 293A cells. Recombinant virus was purified by isolation of an individual viral plaque on 293A cell monolayers.

### Identification of G and N proteins by immunofluorescence assay

Protein expression (i.e., the G ectodomain with signal peptide, or the G or N protein) was identified by immunofluorescence assay (IFA) using specific monoclonal antibodies. The 293A cells infected with Ad-0910Gsped, Ad-0910G, and Ad-0910 N were fixed in cold acetone for 20 min at 2 days post-infection and allowed to air dry completely. The fixed cells were rinsed three times with PBS (pH 7.2), then incubated for 1 h at 37 °C with a 1:500 dilution of monoclonal antibodies against G or N protein of RABV (produced in our lab) in PBS. The cells were rinsed three times with PBS and incubated with a 1:200 dilution of goat anti-mouse IgG conjugated to fluorescein isothiocyanate (KPL, Gaithersburg, MD, USA) in PBS for 1 h at 37 °C. The cells were rinsed three times with PBS and fluorescence was examined under ultraviolet (UV) light illumination with a Nikon microscope (Nikon, Japan).

### Identification of G and N proteins by western blot

Lysates of 293A cells infected with Ad-0910Gsped, Ad-0910G, and Ad-0910 N were separated by NuPAGE 4–12% Bis-Tris gels and transferred to polyvinylidene difluoride (PVDF) membranes (Life technologies). Mock-infected 293A cells and NG108–15 cells infected with the ERA strain were used as negative and positive controls, respectively. The *Xpert*2 Prestained Protein Marker® (GenDEPOT, Barker, TX, USA) was used for identification of G and N proteins molecular weights in MES (red band, 72 kDa; blue band, 57 kDa) and MOPS (red band, 70 kDa; blue band, 53 kDa) Buffer, respectively. Each membrane was placed in blocking solution [5% fat-free milk in Tris-buffered saline-Tween (TBS-T)] at room temperature for 1 h, then incubated with a 1:1000 dilution of monoclonal antibodies against G or N proteins of RABV (produced in our lab) in TBS-T at 4 °C overnight. The membrane was rinsed in TBS-T for 1 h then incubated with goat anti-mouse IgG conjugated with alkaline phosphatase (KPL, Gaithersburg, MD, USA) at a dilution of 1:2000 in TBS-T for 2 h at room temperature. The membrane was rinsed with TBS-T for 1 h and a positive band on the membrane was detected using a colorimetric phosphatase substrate (KPL, Gaithersburg, MD, USA).

### Test of the efficacy of recombinant adenoviruses via intramuscular vaccination in mice

The titer of each recombinant adenoviral stock was determined by infecting 293A cells with 10-fold serial dilutions in the 96 well plates. The cytopathic effect in each well was observed, and the 50% endpoint titers were calculated by the Reed and Muench method and described as TCID_50_/mL. The culture supernatants from Ad-0910Gsped, Ad-0910G, and Ad-0910 N contained titers of 10^8.0^, 10^8.0^, and 10^7.7^ TCID_50_/mL, respectively. Fifty 4-week-old BALB/c mice were grouped randomly into 5 groups (ten per group) as follows: group 1, inoculation with Ad-0910G; group 2, mixed inoculation with Ad-0910G and Ad-0910 N at a 1:1 ratio; group 3, inoculation with Ad-0910Gsped; group 4, mixed inoculation with Ad-0910Gsped and Ad-0910 N at a 1:1 ratio; and group 5, mock-infected mice (Table 1). Groups 1 to 4 were inoculated with 0.2 mL of individual or mixed virus on day 0 by the intramuscular (IM) route. Mice in group 5 were inoculated with an equal volume of PBS. Five mice from each group were challenged intramuscularly with 100 μL of CVS-N2c (25 LD_50_/0.1 mL) at 21 days following vaccination with recombinant adenoviruses. The remaining five mice in each group were challenged with 30 μL of CVS-N2c (25 LD_50_/0.03 mL) by the intracranial (IC) route. After challenge, the survival of mice was checked daily for at least 3 weeks. Mice were euthanized when they developed clinical signs of rabies or 3 weeks post-challenge. This animal experiment was approved by the Animal Care and Use Committee of the Animal and Plant Quarantine Agency (QIA) (approval no. 2013–163).

**Table 1:** Groups of mice immunized with vaccines by intramuscular and oral route

IM group^a^	1		2		3		4		5	
Vaccine^b^	Ad-0910G		Ad-0910G + Ad-0910 N		Ad-0910 Gsped		Ad-0910 Gsped + Ad-0910 N		PBS	
Vaccine volume	0.2 mL		0.2 mL (0.1 + 0.1 mL)		0.2 mL		0.2 mL (0.1 + 0.1 mL)		0.2 mL	
No. of vaccination	1		1		1		1		1	
Challenge route^c^	IM	IC	IM	IC	IM	IC	IM	IC	IM	IC
Oral group^d^	6		7		8		9		10	
Vaccine^e^	V-RG		Ad-0910G + Ad-0910 N		V-RG		Ad-0910G + Ad-0910 N		PBS	
Vaccine volume	0.1 mL		0.1 mL (0.05 + 0.05 mL)		0.1 mL		0.1 mL (0.05 + 0.05 mL)		0.1 mL	
No. of vaccination^f^	1		1		2		2		1	
Challenge route^g^	IC		IC		IC		IC		IC	

^a^Fifty 4-week-old mice were randomly grouped into 5 groups (ten/group) for intramuscular (IM) vaccination

^b^The titers of Ad-0910G, Ad-0910Gsped and Ad-0910 N were 10^8.0^ TCID_50_/mL, 10^8.0^ TCID_50_/ mL and 10^7.7^ TCID_50_/mL, respectively

^c^Immunized mice of each group were divided into two subgroup and intramuscularly (IM) and intracranially (IC) challenged with CVS-N2c at 21 days following inoculation of recombinant adenoviruses

^d^Thirty 4-week-old mice were divided into 4 groups (five/group) and control group (ten/group) for oral vaccination

^e^The titers of V-RG (Raboral V-RG®, Merial), Ad-0910G and Ad-0910 N were 10^7.8^ TCID_50_/mL, 10^8.0^ TCID_50_/mL and 10^7.7^ TCID_50_/mL, respectively

^f^Mice in the groups 6 and 7 were vaccinated with 0.1 ml of V-RG vaccine or mixed virus (Ad-0910G + Ad-0910 N) via the oral route only once. Mice in the groups 8 and 9 were vaccinated orally once again a week after initial vaccination

^g^All mice were challenged with CVS-N2c by the IC route 14 days post-vaccination

### Efficacy test of recombinant adenoviruses via oral vaccination in mice

Twenty 4-week-old BALB/c mice were divided randomly into 4 groups (five per group): group 6, V-RG (Raboral V-RG®, Merial) (1); group 7, Ad-0910G + Ad-0910 N (1); group 8, V-RG (2); and group 9, Ad-0910G + Ad-0910 N (2). Mice in the V-RG (1) and Ad-0910G + Ad-0910 N (1) groups were vaccinated with 0.1 ml of V-RG vaccine or mixed virus (Ad-0910G + Ad-0910 N) via the oral route only once. Mice in the V-RG (2) and Ad-0910G + Ad-0910 N (2) groups were vaccinated orally once again a week after initial vaccination. Ten of the control mice (group 10) were inoculated with an equal volume of PBS (Table 1). All mice were challenged with 30 μL of CVS-N2c (25 LD_50_/0.03 mL) by the IC route 14 days post-inoculation. After challenge, the survival of mice was checked daily for 2 weeks. This animal experiment was approved by the Animal Care and Use Committee of the Animal and Plant Quarantine Agency (QIA) (approval no. 2014–206).

### Statistical analysis

Log-rank tests were performed for statistical analysis among groups in the animal experiments using GraphPad Prism software (Version 7.0). Statistical significance was defined as *P*-values less than 0.05.

## Results

### Construction of recombinant adenoviruses

**Fig. 1:**
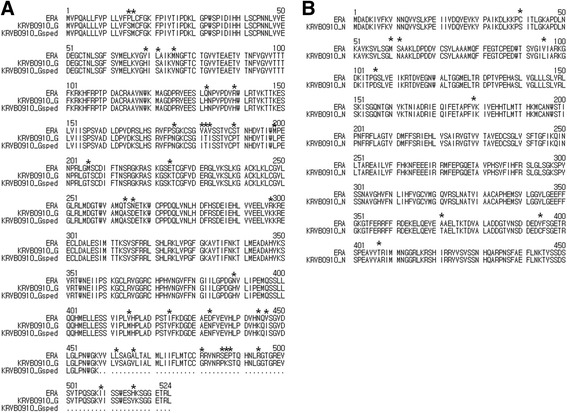
Comparisons of amino acids sequences of G gene (**a**) and N gene (**b**) of Korean street rabies virus (KRVB0910) isolated from RABV-infected cattle with those of reference vaccine strain, ERA (AB781935). The dots indicate amino acids of transmembrane and cytoplasmic domains of G protein, which were removed for construction of Ad-0910Gsped. The asterisks (*) indicate different amino acids between KRVB0910 and ERA strain

The amino acids sequences of G and N gene of Korean street rabies virus (KRVB0910) isolated from RABV-infected cattle were respectively different at 33 and 9 sites in comparison with those of reference vaccine strain, ERA (AB781935) (Fig. 1). The amino acids at positions 459 to 524 of G protein, transmembrane and cytoplasmic domains were excluded from the construct of recombinant adenovirus Ad-0910Gsped for expression of sped (Fig. 1a). We constructed recombinant human adenoviruses (Ad-0910G and Ad-0910 N) expressing the complete G and N proteins of KRVB0910 in a previous report [25], and recombinant adenoviruses (Ad-0910Gsped) expressing the sped of G protein was constructed in this study. Insertion of the sped of the G gene and complete G and N genes into recombinant adenoviruses was confirmed by PCR and DNA sequencing of viral stocks (data not shown). The titers of recombinant Ad-0910Gsped, Ad-0910G, and Ad-0910 N reached 10^8.0^, 10^8.0^, and 10^7.7^ TCID_50_/mL, respectively. Icosahedral capsids typical of adenovirus were observed in 293A cells infected with each recombinant adenovirus using electron microscopy (Data not shown).

### Expression of sped of G protein and complete G and N proteins

**Fig. 2:**
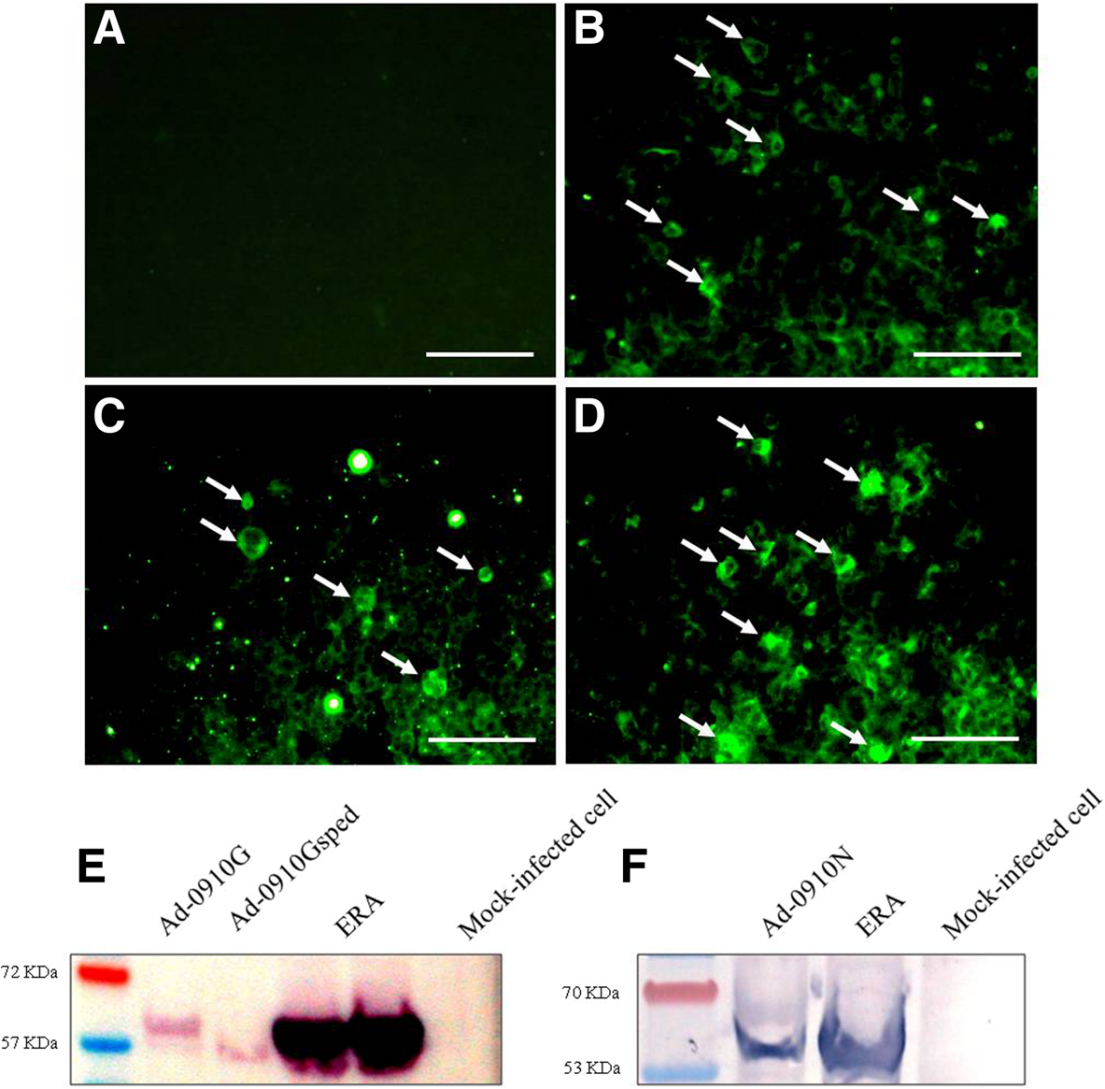
Immunofluorescence assay (IFA) and Western blot analysis for identification of G and N proteins expressed from recombinant adenoviruses. The IFA was performed in mock-infected 293A cells (**a**) and cells infected with recombinant adenoviruses Ad-0910 N (**b**), Ad-0910G (**c**), and Ad-0910Gsped (**d**). Cells were fixed 2 days post-infection and subjected to antibody staining using RABV G and N protein-specific antibodies. Specific fluorescence (*arrows*) was detected in 293A cells infected with each recombinant adenovirus. *Bars*, 100 μm. Western blot analysis was conducted using 293A cell lysates infected with Ad-0910G, Ad-0910Gsped and Ad-0910 N, and monoclonal antibodies against the G or N protein of RABV. (**e**) Lane 1, 293A cell lysates infected with Ad-0910G; lane 2, 293A cell lysates infected with Ad-0910Gsped; lanes 3 and 4, NG108–15 cell lysates infected with the ERA strain; lane 5, mock-infected 293A cell lysates, (**f**) lane 1, 293A cell lysates infected with Ad-0910 N; lane 2 NG108–15 cell lysates infected with the ERA strain; lane 3, mock-infected 293A cell lysates

Expression of the sped of G protein or complete G or N proteins of RABV was analyzed by IFA. 293A cells were fixed 2 days post-infection and subjected to antibody staining using RABV G and N protein-specific antibodies for IFA. The 293A cells infected with each recombinant adenovirus produced specific fluorescence, while no specific fluorescence appeared in the mock-infected 293A cells (Fig. 2a-d). The expression of the recombinant proteins was confirmed using RABV G and N protein-specific monoclonal antibodies in Western blot analysis. The positive bands of complete G protein and truncated sped G protein without the transmembrane and cytoplasmic domains were observed on the membrane with monoclonal antibodies against RABV G protein (Fig. 2e). The band representing recombinant N protein was detected in the 293A cell lysates infected with Ad-0910 N using RABV N protein monoclonal antibodies (Fig. 2f). Cell culture of the ERA strain produced bands in both G and N protein tests, whereas there were no bands in the mock-infected 293A cell lysates (Fig. 2e and f). These results indicated that the sped of G protein, and complete G and N proteins of RABV were indeed expressed in the cells infected with recombinant adenoviruses.

### Protection induced by recombinant adenoviruses via intramuscular vaccination in mice

**Fig. 3:**
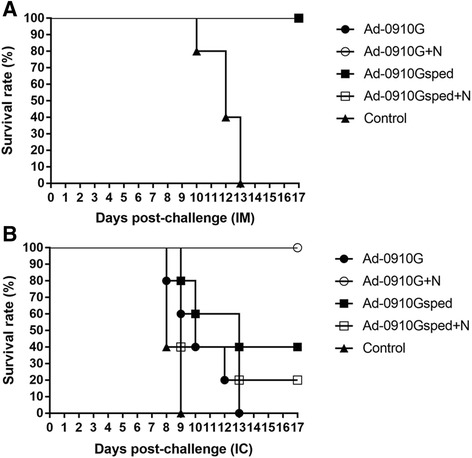
Survival rates of mice immunized with recombinant adenoviruses expressing RABV G and N protein after RABV CVS-N2c challenge. Mice in each group were inoculated with 0.2 mL of each virus (Ad-0910G and Ad-0910Gsped) or mixed virus (Ad-0910G + Ad-0910 N and Ad-0910Gsped + Ad-0910 N) by the intramuscular route. Mice in the control group were inoculated with an equal volume of PBS. Five immunized mice from each group were challenged intramuscularly (IM) with 30 μL of CVS-N2c (25 LD_50_/0.03 mL) 21 days following inoculation of recombinant adenoviruses (**a**). The remaining five mice in each group were challenged via the intracranial (IC) route (**b**). After challenge, the survival of mice was checked daily for 3 weeks

To assess the protection induced by recombinant adenoviruses expressing the sped of G protein (Ad-0910Gsped) and complete G protein (Ad-0910G) in combination with recombinant adenovirus expressing N protein (Ad-0910 N) against challenge with the lethal rabies virus CVS-N2c, fifty BALB/c mice were grouped randomly into 5 groups and immunized intramuscularly with recombinant adenoviruses as shown in Table 1. Immunized mice from all groups except PBS control group exhibited complete (100%) protection against RABV challenge by the IM route (Fig. 3a). The survival rate (100%) of single injection groups of Ad-0910G and Ad-0910Gsped, and combined administration groups with Ad-0910 N was significantly higher than that of mice in the control group (*P* < 0.0001, log rank test). In the IC challenge test, the group of Ad-0910G vaccination did not show protection against viral challenge, whereas the group which was immunized with mixed Ad-0910G and Ad-0910 N viruses showed 100% protection (Fig. 3b). Groups of Ad-0910Gsped, and Ad-0910Gsped with Ad-0910 N exhibited 40 and 20% protection against IC challenge of lethal RABV (Fig. 3b), respectively. The survival rate of mice after combined administration of Ad-0910G and Ad-0910 N was significantly higher than that of mice in the single injection groups with Ad-0910G and Ad-0910Gsped, respectively, (*P* = 0.0018 and 0.0494, log rank test). There was a significant difference when comparing combined administration of Ad-0910G and Ad-0910 N with that of Ad-0910Gsped and Ad-0910 N (*P* = 0.0126, log rank test).

### Protection of recombinant adenoviruses via oral vaccination in mice

**Fig. 4:**
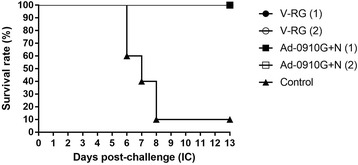
Survival rates of mice immunized with Raboral vaccinia-rabies glycoprotein (V-RG®) vaccine and mixed virus (Ad-0910G + Ad-0910 N) by the oral route after RABV CVS-N2c challenge. Mice in the V-RG (1) and Ad-0910G + Ad-0910 N (1) group were inoculated with 0.1 mL of VRG vaccine and mixed virus (Ad-0910G + Ad-0910 N) via the oral route only once. Mice of V-RG (2) and Ad-0910G + Ad-0910 N (2) group were inoculated orally again one week after initial inoculation. Mice in the control group were inoculated with an equal volume of PBS. All mice were challenged with 30 μL of CVS-N2c (25 LD_50_/0.03 mL) by the intracranial (IC) route 14 days following vaccination. After challenge, the survival of mice was checked daily for 2 weeks

The mixed virus (Ad-0910G + Ad-0910 N) and Raboral V-RG® vaccine used as a positive control vaccine were administered orally to 5 mice in each group to investigate the protection efficacy of oral vaccination with mixed virus (Ad-0910G + Ad-0910 N), which showed 100% protection against IM and IC challenge of lethal RABV via IM vaccination. The mice in the mixed virus (Ad-0910G + Ad-0910 N) group had a 100% survival rate, the same as the Raboral V-RG vaccine group, in both single and two-dose oral vaccination, whereas mice in the control group had only a 10% survival rate after challenge with lethal RABV (Fig. 4). The survival rate after oral administration of the combined Ad-0910G and Ad-0910 N and Raboral V-RG vaccine was significantly higher than that of mice in the negative control group (*P* < 0.0001, log rank test). These results indicate that mixed virus (Ad-0910G + Ad-0910 N) in both IM and oral vaccinations can confer complete protection against the rabies virus.

## Discussion

G protein plays a significant role in cell-to-cell spread, transport, replication, pathogenicity, and immunogenicity of RABV [26]. The pathogenic street RABV strain induces a low level of apoptosis/necrosis and virulence in infection due to its lower replication rate, reduced G protein expression, and differences in the amino acid sequence of its G protein compared to other pathogenic RABV strains, resulting in evasion from immune response [26, 27]. The street virus and a recombinant fixed virus expressing the G protein from the street virus exhibited weaker innate immune and inflammatory responses compared to typical RABV [28]. Currently, most recombinant viruses created in various vector systems carry G genes from attenuated RABV strains instead of the pathogenic street RABV strain [10, 11, 17, 19]. Therefore, there were not enough reports about protection effects of recombinant viruses expressing the proteins of pathogenic street virus against RABV. We constructed recombinant human adenoviruses (Ad-0910G and Ad-0910 N) expressing the complete G and N protein of Korean street RABV in the previous study [25], and recombinant adenovirus (Ad-0910Gsped) expressing the sped of G protein in this study. The combined injection of Ad-0910G and Ad-0910 N containing titer of 10^8.0^ TCID_50_/mL was safe and induced respectively 100% and 70% immunization via IM and oral administration in Korean raccoon dogs [25]. In order to compare and evaluate the protective immunity induced by a single and combined vaccination of recombinant adenoviruses, three kinds of recombinant adenoviruses (Ad-0910Gsped and Ad-0910G with or without Ad-0910 N) were administered before IM and IC challenge in mice in this study. Single injection of recombinant adenoviruses expressing complete and truncated G protein as the main immunogen and a combination of recombinants expressing G and N proteins protected all mice that were later challenged intramuscularly with lethal RABV. It appears that the G and N proteins of the pathogenic street RABV strain can generate protective immunity against peripheral infection.

However, different results were found from IC challenge. Single immunization of Ad-0910G and Ad-0910Gsped and immunization with a combination of Ad-0910Gsped and Ad-0910 N induced low protection (0, 40, and 20%, respectively), whereas combined administration of Ad-0910G and Ad-0910 N rendered all mice capable of surviving IC challenge with lethal RABV. Combined complete G and N proteins from street RABV exhibited greater protection against IC exposure to lethal RABV in comparison to single complete G, truncated G protein, and a combination of truncated G protein and N protein. These results suggest that complete G protein can be a more effective immunogen than truncated G protein with the transmembrane and cytoplasmic domains removed, and that the combination of G and N proteins confers improved efficacy versus single administration of G protein in tests of vaccines using proteins from street RABV.

The soluble form of G protein (Gs) lacking 58 amino acid residues, including most of the transmembrane domain and the entire cytoplasmic domain at the carboxy terminus, showed poor protective immune response compared to intact viral G protein [29]. A previous study reported that the cytoplasmic domain is essential for making the 1–30-44 epitope-positive mature form [30]. In a recent study, soluble truncated recombinant G protein showed similar immunogenicity to full-length G protein in rabbits [31]. The amino acids at positions 459 to 524 of the transmembrane and cytoplasmic domains of G protein were excluded from expression in the signal peptide and ectodomain (sped) of G protein in the recombinant adenovirus Ad-0910Gsped in this study. The complete and truncated G protein showed the same protective capacity of single administration against IM exposure to RABV, but induced different protective activity in a combination vaccination with N protein against IC challenge. Therefore, further studies are needed into whether the carboxy terminus of RABV G protein is a critical factor in protective immunity against pathogenic RABV.

Although G protein is the main immunogen for protection against RABV, some research has demonstrated that N protein or RNP can also induce protective immunity against a subsequent challenge with RABV. N protein has been expressed in various expression systems, including vaccinia virus, insect cells, plants, and silkworm larvae, and its protective ability has been tested in a challenge experiment [2, 7–9]. Mice vaccinated intradermally with a Copenhagen vaccinia virus recombinant expressing the N protein of the CVS strain showed a protective response against peripheral RABV challenge [2]. Inoculation with N protein expressed in insect cells increased the production of Nab prior to a booster vaccination, elicited T- and B-cell responses, and protected against a lethal RABV challenge in mice [7]. Fifty percent of mice immunized intraperitoneally with tomato extract containing N protein survived a peripheral virus challenge [8]. IM immunization with N protein expressed in silkworm larvae contributed to 90% protection in mice challenged with an intracerebral inoculation of the CVS strain [9]. Interestingly, the incubation period of RABV was reduced in dogs and some dogs sickened then recovered without supportive treatment when dogs were vaccinated with vaccinia virus recombinants expressing RABV N protein and then challenged with a street rabies virus [4]. Collectively, these works indicate that N protein contributes protection against RABV infection in the absence of Nab-inducing G protein.

A few studies have mentioned the protective efficacy of combination administration of RABV G and N protein or RNP. Striped skunks immunized by oral inoculation with a raccoon poxvirus recombinant containing G protein of Challenge Virus Standard (CVS) and a mixture of raccoon poxvirus RABV G and N protein recombinants showed 30% and 20% survival rates, respectively, against IM challenge with street RABV [32]. Dogs immunized with the vaccinia virus recombinant expressing G and N proteins of CVS were protected against IM challenge with a street RABV [4]. A liposome containing purified RNP and G protein of ERA and CVS confers a 13-fold higher protective response than a G protein-only liposome against an intracerebral challenge in mice [6]. Although our previous results showed that oral vaccination with combined virus (Ad-0910G and Ad-0910 N expressing G and N protein from pathogenic street RABV) induced lower levels of Nab (0.17 ~ 0.5 IU/mL) than those (0.5 ~ 13.7 IU/mL) of IM route vaccination in raccoon dogs [25], an oral administration in mice generated the same complete protection against lethal RABV as the commercial oral vaccine, Raboral V-RG®, in response to IC challenge in this study. In IM route study, combined vaccination protected all mice against an IC challenge with lethal RABV CVS-N2c, whereas single vaccination of Ad-0910G failed to confer protective immunity against IC challenge.

The mechanism of protective immunity induced by N protein is still not fully understood. However, N protein is the main constituent of RNP that activates functional B and T-helper cells, resulting in protection against viral infection [6, 7, 33, 34]. Along with N protein, immunization with a purified RNP in complete Freund’s adjuvant conferred protective immunity against lethal peripheral RABV challenge in mice and raccoons [6]. RNP stimulates RABV-specific T-helper cells that facilitate the action of Nab-producing B cells and also induce cytolytic T cells to eliminate the RABV [6, 7, 33]. Previous studies revealed that the G proteins of fixed or street RABVs have antigenic variations and this variation could cause occasional failures of post-exposure protection against RABV [9, 35]. RNP might induce protective immunity against heterologous lyssaviruses and may be effective in post-exposure therapy by inducing immune factors such as cytolytic T cells [6]. Additionally, N protein was related to RABV pathogenicity in adult mice in a comparison between nonlethal strain Ni-CE and virulent strain Nishigahara [36]. The virulent RABV N protein suppresses induction of interferon and chemokines in neuroblastoma cells and is important for inhibition of innate immune response, viral propagation, and spread of pathogenicity in the mouse brain [37, 38]. Thus it is assumed that vaccination of mice in our study with street RABV N protein contributed to their survival after IC exposure to RABV. Although the immunological and pathogenetic basis of protection after vaccination with N protein should be studied thoroughly, it has been shown that N protein plays a momentous role in inducing protective activity against rabies infection.

## Conclusions

In this study, the Ad-0910G virus, expressing the complete G protein of Korean street RABV, was more immunogenic than Ad-0910Gsped, which expressed a truncated G protein with the transmembrane and cytoplasmic domains removed. A combination of Ad-0910G and Ad-0910 N conferred improved immunity against IC challenge compared to single administration of Ad-0910G and led to complete protection in oral vaccination. Further studies are needed to investigate whether recombinant adenoviruses expressing the G and N proteins of Korean street RABV are able to induce protective immunity against various RABVs, and the humoral and cellular immune response after inoculation of recombinant adenoviruses. However, the results of this study suggest that combination viruses (Ad-0910G + Ad-0910 N) are useful new IM and oral vaccine candidates.

## Abbreviations

CVS: Challenge virus standard
DMEM: Dulbecco’s modified eagle’s medium
ERA: Evelyn-rokitnicki-abelseth
G: Glycoprotein
Gs: Soluble form of G protein
IC: Intracranial
IFA: Immunofluorescence assay;
IM: Intramuscular
L: Large polymerase protein
LD_50_: Median lethal dose
M: Matrix protein
N: Nucleoprotein
Nab: Neutralizing antibody
NEAA: Non-essential amino acids
P: Phosphoprotein
RABV: Rabies virus
RNP: Ribonucleoprotein
Sped: Signal peptide and ectodomain
V-RG: Vaccinia-rabies glycoprotein
